# What Drives Vaccine Uptake?—Investigating the Application of the Health Belief Model Through a Longitudinal Cohort Study During the COVID-19 Pandemic in Victoria, Australia

**DOI:** 10.3390/vaccines13101021

**Published:** 2025-09-30

**Authors:** Anita Voloshin, Aimée Altermatt, Anna Wilkinson, Katherine B. Gibney, Sophie Hill, Jessica Kaufman, Rebecca E. Ryan, Margie Danchin, Alisa Pedrana, Margaret E. Hellard, Katherine Heath

**Affiliations:** 1 Burnet Institute, 85 Commercial Road, Melbourne, VIC 3004, Australiakatie.heath@burnet.edu.au (K.H.); 2 School of Public Health and Preventive Medicine, Monash University, 533 St Kilda Road, Melbourne, VIC 3004, Australia; 3 Peter Doherty Institute for Infection & Immunity, The University of Melbourne, 792 Elizabeth Street, Melbourne, VIC 3000, Australia; 4 Centre for Health Communication and Participation, La Trobe University, Bundoora, VIC 3086, Australia; 5 Murdoch Children’s Research Institute, 50 Flemington Road, Parkville, VIC 3052, Australia; 6 Faculty of Medicine, Dentistry and Health Sciences, The University of Melbourne, Parkville, VIC 3010, Australia; 7 Department of Paediatrics, University of Melbourne, Parkville, VIC 3010, Australia; 8 Department General Medicine, The Royal Children’s Hospital Melbourne, Parkville, VIC 3052, Australia; 9 Department of Epidemiology and Preventive Medicine, Monash University, 533 St Kilda Road, Melbourne, VIC 3004, Australia; 10 Doherty Institute and School of Population and Global Health, University of Melbourne, Parkville, VIC 3010, Australia; 11 Department of Infectious Diseases, The Alfred Hospital, 55 Commercial Road, Melbourne, VIC 3004, Australia

**Keywords:** health belief model, COVID-19 vaccine uptake, vaccine hesitancy, longitudinal cohort study, risk perception, pandemic response, public health communication, behavioral health theory

## Abstract

Background/Objectives: Understanding the psychological determinants of vaccine uptake is critical for effective public health strategies, particularly during prolonged pandemics. The Health Belief Model is widely used to examine vaccine behavior, yet its applicability in longitudinal and policy-intensive contexts remains underexplored. This study assessed how two core Health Belief Model constructs—perceived severity of and susceptibility to COVID-19—related to vaccine intentions and uptake over time, and how these perceptions varied by demographic characteristics. Methods: Data came from Optimise, a longitudinal cohort study of adults in Victoria, Australia, conducted between September 2020 and August 2022. Perceived severity of and susceptibility to COVID-19 were measured monthly, alongside COVID-19 vaccine intentions and uptake. Generalized Estimating Equations evaluated associations between these two Health Belief Model constructs and vaccine outcomes over time. Separate models identified demographic predictors of perceived severity and susceptibility. Results: Perceived severity of COVID-19 was positively associated with intention to receive further COVID-19 vaccine doses (OR = 2.53, 95% CI: 1.26–5.07) and the total vaccine doses received (OR = 2.74, 95% CI: 1.58–4.76), with these associations changing over time as vaccine mandates were lifted and the pandemic context evolved. Perceived susceptibility to COVID-19 showed no significant associations with vaccine outcomes. Older age, presence of a chronic health condition, and lower employment status was associated with higher perceived severity. In contrast, perceived susceptibility was higher among high-income earners but lower among older adults and the unemployed. Conclusions: The predictive value of two Health Belief Model constructs was context- and time-dependent. Perceived severity consistently predicted vaccine uptake once mandates were lifted, while susceptibility did not. Our findings highlight the importance of context-sensitive behavioral frameworks when designing vaccine promotion strategies during extended public health crises.

## 1. Introduction

The COVID-19 pandemic presented a major public health challenge, with vaccination as a key strategy for preventing severe disease and mitigating transmission. To maximize vaccine uptake, public-health programs must be informed by robust behavioral theory that explains why people decide to vaccinate, and how those motives change as a pandemic unfolds.

**Figure 1:**
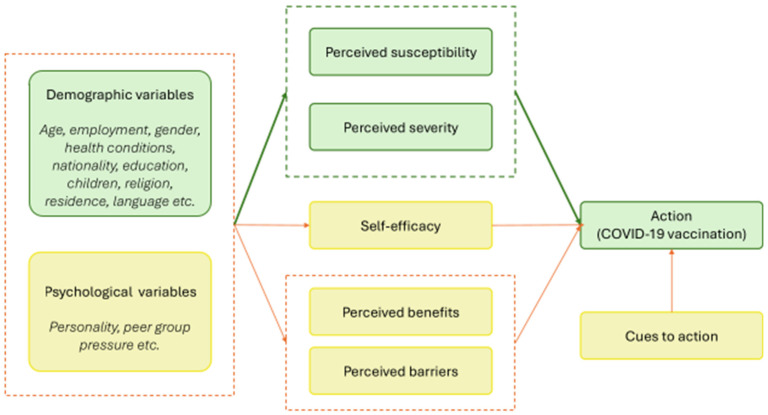
The Health Belief Model, illustrating how perceptions of susceptibility, severity, benefits, barriers as well as self-efficacy and cues to action influence COVID-19 vaccination. Components analyzed as part of this research are highlighted in green. Dashed lines group related constructs. Arrows represent the direction of influence between constructs or groups of constructs.

The Health Belief Model is a longstanding framework to understand the cognitive drivers of an individual’s intention to uptake particular health-centric behaviors [1]. It proposes six determinants: perceived severity, perceived susceptibility, perceived benefits, perceived barriers, self-efficacy, and cues to action (Figure 1). The model has been widely applied across a range of public health contexts, including smoking cessation, breast cancer screening and, more recently, COVID-19 vaccination. Although the model is well accepted in contemporary literature, it has been criticized for its variable explanatory power and its limited attention to social and environmental influences [2]. Nonetheless, it remains valued for its simplicity and cross-context relevance [3].

The application of the Health Belief Model framework to prospectively understand vaccine uptake has been undertaken across a range of global settings, including Asia, North America and Europe. A systematic review has indicated that perceived susceptibility and severity were negatively associated with COVID-19 vaccine hesitancy across a range of global settings [4]. In other words, people who perceived themselves as more susceptible to COVID-19, or who viewed the disease as more severe, were less hesitant to vaccinate. In addition, perceptions can be influenced by trusted information sources, including media, government communications, and social networks [5,6]. Despite its widespread geographical application in understanding COVID-19 vaccine hesitancy, most evidence comes from cross-sectional studies, and no research has examined the Health Belief Model longitudinally in Australia.

**Figure 2:**
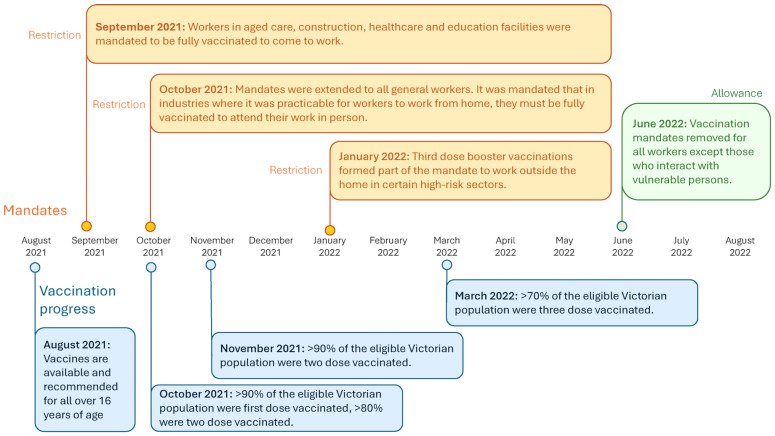
Timeline of COVID-19 mandates and restrictions in Victoria, Australia August 2021–August 2022.

Victoria, Australia’s second-most populous state, offers a compelling opportunity to explore the drivers of vaccine uptake. After initially maintaining low case numbers through stringent border controls and the world’s longest cumulative lockdowns, Victoria shifted in early- to mid-2021 to a rapid vaccination strategy, as shown in Figure 2 [7,8,9,10,11,12,13,14]. One-dose coverage surpassed 95% within 12 months, propelled by intensive public messaging and mandates, including vaccination thresholds requiring 70% full vaccination to end lockdown [13,15,16]. At the height of the pandemic, vaccine requirements in Victoria were among the strictest in Australia, covering healthcare, education, construction, aged care and hospitality. From late October 2021, entry into hospitality venues was restricted to those with proof of full vaccination. In this context, uptake of initial, second and third doses occurred under restricted autonomy, limiting the applicability of Health Belief Model assumptions about free choice. For this reason, vaccine behavior may be most meaningfully assessed for doses beyond the third, when mandates no longer applied.

In mandate-intensive contexts such as Victoria, interpreting vaccine behavior through the Health Belief Model is challenging, since the model assumes individual choice based on perceived risk and benefit. Mandates may override these mechanisms, meaning that Health Belief Model constructs should be interpreted with care and may be most meaningful for uptake beyond the third dose, when mandates were lifted.

Following the removal of mandates for COVID-19 vaccination June 2022, uptake of booster doses (three or more doses) has waned, highlighting the need to examine how individual risk perceptions evolve in the absence of external requirements, particularly as the pandemic response shifts from acute crisis to endemic management [17]. Declining vaccination rates remain a policy concern, reflected in the Australian Government’s COVID-19 Response Inquiry (September 2023) which called for research into the “broad decline in COVID-19 vaccination, especially among priority populations” [18].

Data collected as part of the Optimise study, a longitudinal cohort in Victoria between September 2020 and August 2022, provides a unique opportunity to examine vaccine intention and uptake in Victoria over time. Leveraging monthly data from Optimise, this study applies the Health Belief Model to pandemic vaccine behavior longitudinally. By repeatedly measuring perceptions of severity and susceptibility alongside vaccination outcomes, we test whether the association of these constructs with vaccine intention and behaviour shifted across the pandemic.

This study had two aims: (1) to assess how Health Belief Model constructs, specifically perceived severity and susceptibility, are associated with COVID-19 vaccination intentions and uptake over time, and whether these associations evolved across different phases of the pandemic; and (2) to investigate the demographic factors associated with perceived severity and susceptibility.

By focusing on two key Health Belief Model constructs longitudinally, the study provides timely evidence to inform future vaccination programs and to clarify the conditions under which the Health Belief Model has explanatory value.

## 2. Materials and Methods

### 2.1. Study Design and Participants

The Optimise study was a longitudinal cohort study held in Victoria, Australia that surveyed participants on their attitudes and behaviors during the COVID-19 pandemic. Data were collected between September 2020 and August 2022, with continuous recruitment from September 2020 to December 2021. Participants were eligible if they were over 18, lived in Victoria, were able to provide informed consent, had a valid and accessible email or phone number. Participants were ineligible if they were in hospital or too unwell to participate at the time of recruitment [19]. People who were at an elevated risk of COVID-19 or were to be disproportionately impacted by public health orders were targeted for recruitment [19].

The survey could be self-completed online or administered over the phone by research staff in English, Dinka, Arabic and/or Mandarin. There were four components to the data collection: (1) a baseline survey completed once at the beginning of data collection, (2) a daily diary for 14 days once at the beginning of data collection, (3) monthly follow up surveys, and (4) follow-up diaries that were completed four times a month. Each participant was remunerated with online gift vouchers for their time ($35 for the baseline survey, $15 for at least 10 baseline daily diary entries, $2.50 per follow-up daily diary entry and $25 for each monthly follow up survey) [19].

The study was approved by Alfred Health Ethics (approval number 333/20), and the protocol has been published [19].

### 2.2. Outcomes

The primary outcomes for the analyses in this study were: perceived severity of COVID-19, perceived susceptibility to COVID-19, intention to have a first COVID-19 vaccine dose, intention to have further COVID-19 vaccine doses and, self-reported number of COVID-19 vaccines doses received. To address aim one, intention to have a first COVID-19 vaccine, intention to have further COVID-19 vaccines and COVID-19 vaccine doses were dependent variables in three separate models. Perceived severity and susceptibility to COVID-19 were explanatory variables alongside time. For aim two, perceived severity and susceptibility to COVID-19 were dependent variables, and demographic factors were explanatory variables.

From May 2022, the survey was amended to capture evolving vaccine attitudes and behaviors; thus, while intention to have a first dose of COVID-19 vaccine was captured for the entire study period (September to August 2022), data on intention to have further doses and number of doses received were only collected after May 2022.

We limited the scope of our analysis to two drivers of COVID-19 vaccination in the Health Belief Model, perceptions of severity and susceptibility, because we had the most robust longitudinal data for these constructs. The other constructs driving vaccine uptake, as described by the Health Belief Model (Figure 1) were outside the scope of this analysis.

All primary outcomes were recoded as binary variables (see Table 1) to improve interpretability and ensure model stability, and categories for both primary outcomes and demographics were aligned to other published work from the Optimise study [19,20].

The number of COVID-19 vaccine doses received was collapsed into two categories: ≤3 doses and >3 doses. Although sector-specific mandates applied to healthcare, aged care, and other occupations, there were no mandates for a fourth or subsequent dose for the general population. Therefore, uptake beyond three doses is considered to primarily reflect voluntary, intrinsic decision-making

Participant responses “Prefer to not to say” and “Other” were considered missing data and excluded from analysis. For perceived susceptibility, participants could select “not applicable—already been infected with COVID-19” (see Table 1). Although, COVID-19 transmission increased over time, this response did not result in declining data availability as only 181 participant responses out of 11,115 surveys administered had missing data or were not applicable.

### 2.3. Data Analysis

To address Aim 1, we examined how perceived COVID-19 severity and susceptibility influenced vaccine intentions and self-reported uptake over time. Guided by the Health Belief Model, we hypothesized that higher perceived severity of COVID-19 illness and perceived susceptibility to infection would be positively associated with increased vaccine intentions and uptake, but that the strength of these associations would vary over time, reflecting changes in the broader social, epidemiological and policy context of the pandemic.

To test this hypothesis, generalized linear models with Generalised Estimating Equations (GEEs) to account for repeated measures were used. We fit three separate models for the following outcome variables: (i) intention to receive a first COVID-19 vaccine dose (October 2020–September 2021), (ii) intention to receive further doses (May–August 2022) and, (iii) cumulative number of vaccine doses received (May–August 2022). As covariates, each model included time, perceived severity, perceived susceptibility, and their interactions with time. Time was modeled as a categorical variable with interactions, as both outcomes and predictors varied across periods. This allowed us to analyze period-specific associations between perceived susceptibility or severity and the outcomes, capturing contextual, policy-driven, and non-linear dynamics over time.

For outcome (i), first dose intention, time points after intention reached ≥ 95% were excluded due to data sparsity limiting a meaningful analysis. Application of this criterion excluded data after 9 September 2021. In both outcomes (ii), further dose intention, and (iii), doses received, data were considered from May 2022, when questions on these variables were first introduced in the survey.

To specifically explore vaccination decisions made under conditions of choice rather than mandate, we focused our analyses on doses beyond the third vaccine, which in Victoria were no longer mandated and therefore more likely to reflect intrinsic motivation. To account for repeated samples, an autoregressive (AR (1)) correlation structure was used, with decreasing correlation between farther time periods.

The models described above focus on temporal associations between perceived severity and susceptibility with vaccine intention and uptake. Demographic covariates were examined separately as predictors of Health Belief Model constructs, consistent with the study’s aim to assess temporal dynamics rather that to build a fully explanatory model.

To address Aim 2, to examine demographic predictors of (a) perceived COVID-19 severity and (b) perceived susceptibility, we fitted two separate binomial GEE models (logit link). For each model, all combinations of demographic covariates were tested using an AR (1) structure (age, region of residence at baseline, gender identity, existence of a chronic health condition, country of birth, language spoken at home, whether employed as a healthcare worker at baseline, religion, level of education, employment status, and whether children lived in the household). Time was included as a continuous variable because the predictors (demographics) were time invariant, and only the outcomes varied over time. A continuous specification suffices to capture overall associations between outcome variables and demographics, accounts for repeated measures, and reduces model complexity by avoiding separate estimates for each time period.

Models were ranked using the Quasilikelihood under the Independence Model Criterion (QIC), and the model with the fewest covariates within the lowest 10% of QIC values was selected. Time-by-demographic interactions were then added and retained only if they improved model fit. The geepack package was used to conduct the analysis on R v3.6.3. Data analysis was conducted in R version 4.1.1 [21].

## 3. Results

### 3.1. Demographics

The Optimise study included 779 participants across the entire study period of one year and 11 months, the demographics of which are described in Table 2. Across the study period, the number of individuals completing each survey and answering each specific question for analysis fluctuated. These difference in respondents between time periods were due to (a) rolling recruitment and (b) the fact that completing the surveys was voluntary. Additional demographics are included in Appendix A, Table A1.

### 3.2. Perceptions of COVID-19 Severity and Susceptibility over Time

**Figure 3:**
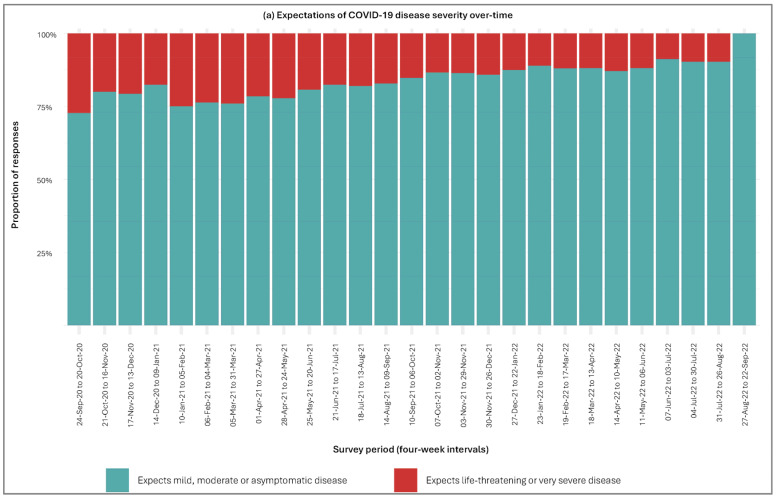

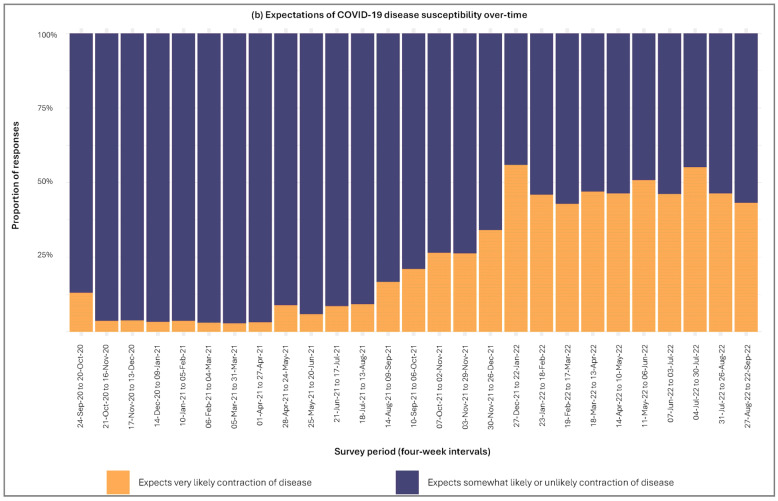
Expectations of COVID-19 disease severity and susceptibility over time. Subfigure (**a**) displays perceived severity of COVID-19 over time. Subfigure (**b**) displays perceived susceptibility to COVID-19 infection over time.

Perceptions of COVID-19 severity and susceptibility fluctuated over time as shown in Figure 3. Throughout the study, most participants expected COVID-19 to be mild, moderate, or asymptomatic, with a smaller proportion perceiving the disease as life-threatening or very severe. The proportion expecting severe disease fluctuated at the onset of the pandemic until early 2021 before it gradually declined over the remainder of the study until September 2022. In contrast, expectations of susceptibility to infection remained relatively stable through 2020 and early 2021, with most respondents reporting that they were somewhat unlikely or unlikely to contract COVID-19. From mid-2021 onwards, however, the proportion of participants who believed they were very likely to contract the virus increased substantially—peaking in late 2021 and early 2022—before declining marginally in subsequent months.

### 3.3. Aim 1: Perceived Severity, Susceptibility and Vaccine Outcomes over Time

For intention to have a first dose of COVID-19 vaccine, time was a significant predictor, with odds of intending to vaccinate increasing notably at later time points (*p* < 0.01). However, neither perceived severity nor susceptibility showed consistent independent effects or significant interactions with time, as shown in Table 3.

In contrast, perceived severity was significantly associated with intention to receive further vaccine doses (OR = 2.53, 95% CI: 1.26–5.07, *p* = 0.01) (Table 4) and having received more than 3 doses (OR = 2.74, 95% CI: 1.58–4.76, *p* < 0.01) (Table 5). The association of perceived severity and the number of doses received was weaker at time T24 (4 July 2022 to 30 July 2022) compared to T22 (11 May 2022 to 6 June 2022), as indicated by a significant interaction term reflecting effect modification by time. The overall associations of perceived susceptibility with further vaccine intention or dose count were not statistically significant (Table 4 and Table 5). Interaction terms with time indicate that the effect may vary at specific time points, although none of these time-specific effects reached statistical significance. A graphical representation of the relationships between perceived severity and susceptibility with vaccine outcomes is given in Appendix B.

### 3.4. Aim 2: Demographic Predictors of Health Belief Model Beliefs

GEE model selection for perceived severity of COVID-19 included age, chronic health condition, employment status, and time (28-day blocks) as covariates. Participants aged 55–64 and 65+ were more than four times more likely to perceive COVID-19 as severe than those aged 18–24. Those with a chronic condition and those not in full-time employment were both over twice as likely to perceive COVID-19 as severe compared to those without a chronic health condition and those in full time employment. Model results are shown in Table 6.

### 3.5. The Demographics of Perceived Elevated Susceptibility to COVID-19

GEE model selection for perceived susceptibility to COVID-19 included age, chronic health condition, employment status, enrolment as a healthcare worker, household income, membership of a religious group, region of residence and time (28-day blocks) as covariates. Participants aged 55–64 and 65+ were more than four times more likely to perceive COVID-19 as severe than those aged 18–24. Despite perceiving COVID-19 as more severe, individuals aged 65+ and individuals not employed perceived themselves as less susceptible to COVID-19 than those aged 18–25 and those in full time employment. Notably, individuals with a household income over AUD$150 k perceived themselves as more than twice as susceptible than those earning under AUD50 k. Model results are shown in Table 7.

## 4. Discussion

This study builds on previous work examining the drivers of Victorians’ vaccine behavior, and adds to a growing body of evidence suggesting that vaccine decision-making is influenced by a complex combination of factors [17,20,22,23]. We examined two constructs of the Health Belief Model (Figure 1)—perceived severity of and susceptibility to COVID-19—to assess their relevance for longitudinal vaccine uptake. Our study had two main findings. First, the explanatory value of widely accepted frameworks, like the Health Belief Model, is dynamic and time-dependent, likely influenced by the policy and environmental context in which individuals are operating. Second, perceived severity of COVID-19 was a more consistent predictor of vaccine intention than perceived susceptibility to the disease.

We did not observe any association between perceptions of COVID-19 severity or susceptibility and intention for the first COVID-19 vaccine dose, which was measured early in the pandemic when vaccines were not yet available and mandates shaped decision-making. However, perceptions of severity were associated with intention for subsequent doses, measured after mandates were lifted and vaccination decisions were more likely to reflect genuine choice rather than compliance. This shift highlights that the predictive value of Health Belief Model constructs can be shaped by time and policy context.

Several factors may explain this finding. One is the time sensitive nature of the survey questions. ‘Intention to have the first dose of the vaccine’ was asked early in the pandemic when vaccines were unavailable and stringent restrictions were in place, meaning the responses given were somewhat hypothetical. ‘Intention to have subsequent doses’ was measured later (from May 2022) after it was clear that multiple regular vaccinations would be required and after restrictions had recently eased. These two time periods had distinctly different policy and epidemiological contexts, with the later decisions arguably more likely to reflect personal perceptions of severity, rather than compliance. This hypothesis is supported by our data and by literature reporting that the Health Belief Model’s predictive value is heavily influenced by the conditions in which it is being applied, and that policy, media and social norms might be key mediating factors on its applicability [24].

Another explanation is that decision-making may differ between initial and subsequent doses. A systematic review found that the perceived severity declined in predictive value for COVID-19 booster dose intention [25]. Although this contrasts with our finding that perceived severity was more associated with subsequent doses, both studies suggest that the influence of Health Belief Model constructs may change depending on prior vaccination history. Given that time, context and vaccination history may all be mediating the applicability of the Health Belief Model to vaccine intention, further investigation is needed to understand under which conditions it is most relevant.

Perceived severity was also significantly associated with the number of vaccine doses received. One explanation is the Health Belief Model may better predict actual behavior than hypothetical intention. Another possible explanation is the characteristics of our study sample, which oversampled those at elevated risk of COVID-19 and its health effects. Participants with pre-existing chronic health conditions reported higher perceived severity of COVID-19, aligning with existing literature suggesting that this group engages in distinct health decision-making processes due to their different risk profile [26]. Similarly, older adults reported higher perceived severity but lower perceived susceptibility, suggesting that those at greater clinical risk may perceive a lower likelihood of infection due to protective behaviors or reduced exposure [27]. Consequently, the observed associations may partly reflect an over-representation of individuals who both perceived COVID-19 as severe and had greater vaccination opportunities due to policy measures, rather than a direct causal link between severity perception and uptake. Further research is needed to examine these relationships across populations and policy contexts, underscoring the challenge of disentangling intrinsic motivation from structural influences.

Perceptions of severity and susceptibility also varied across socioeconomic and demographic groups and were influenced by broader mediators such as vaccine confidence and health literacy. Higher perceived severity among older adults and individuals with chronic conditions likely reflects both clinical risk and greater engagement with health information, whereas perceived susceptibility was higher among high-income earners but lower among older adults and the unemployed, potentially reflecting differences in exposure, protective behaviors, and access to information. Individuals with higher confidence in vaccines and greater understanding of health information may interpret infection risks differently, influencing both perceived severity and subsequent vaccination behavior [28,29]. These patterns illustrate how social determinants and mediating factors shape how individuals interpret and respond to disease risk, highlighting the importance of considering these influences when applying behavioral models such as the Health Belief Model.

The variability of the Health Belief Model’s predictive value across time and demographic subgroups suggests it is best applied to specific contexts rather than entire populations. Prior research similarly shows that health beliefs and vaccine uptake are shaped by structural and temporal factors [30,31,32]. In the case of COVID-19, influencers such as trust in institutions, social norms, and perceived benefits—constructs outside the Health Belief Model—have also been shown to influence vaccine uptake [33,34]. Therefore, while the Health Belief Model remains a valuable framework, our findings suggest it may be best applied contextually and in conjunction with broader behavioral and structural considerations, including whether decisions reflect choice or mandate-driven compliance.

### Limitations

Our cohort intentionally oversampled healthcare workers and priority groups, increasing statistical power for these populations but limiting generalizability. Attrition across survey waves may also introduce bias toward more engaged participants, although high retention rates across the study imply that this effect may be minimal. Participants who responded “prefer not to say” or “don’t know” to survey questions were excluded from some analyses, which may introduce selection bias and limit the generalizability of our findings. However, the proportion of participants responding with these options was small. Not all participants responded at every survey wave; for some months, only a subset of the cohort completed the survey. This variation in response may introduce some selection bias, as findings could disproportionately reflect individuals who remained engaged across multiple waves. However, GEE models allow inclusion of all available responses, focusing on temporal trends rather than individual-level predictions.

## 5. Conclusions

The Health Belief Model is widely used to explain health behavior, but its utility during the COVID-19 pandemic in Victoria proved context-dependent. Our findings show that once mandates were lifted, perceptions of disease severity—but not susceptibility—were consistently associated with vaccine intention and uptake. Our findings highlight the challenges of applying generalized behavioral models in highly dynamic policy environments and the importance of situating them within demographic and structural contexts. Future research should clarify when and for whom the Health Belief Model most accurately predicts vaccine intention and behavior.

## Figures and Tables

**Table 1:** Survey questions included in analysis and the processing that was completed prior to their inclusion.

Overview	Question	Possible Responses	Post-Processing
Intention to have a COVID-19 vaccine	[Before 26 May 2021] If a COVID-19 vaccine was to become available to everyone in Australia, do you think you would have it yourself?	Definitely yes Probably yes Probably not Definitely not Unsure Prefer not to say	‘Definitely yes’ was reclassified as ‘1’ ‘Prefer not to say’ was reclassified as ‘No data’. All other responses were reclassified as ‘0’.
	[After 26 May 2021] Do you think you would have a COVID-19 vaccine?	I have already been vaccinated Definitely yes Probably yes Probably not Definitely not Unsure Prefer not to say	‘I have already been vaccinated’ and ‘Definitely yes’ were reclassified as ‘1’ ‘Prefer not to say’ was reclassified as ‘No data’. All other responses were reclassified as ‘0’.
Intention to have further COVID-19 vaccine doses	Do you think you would have further doses of the vaccine if recommended?	Definitely yes Probably yes Probably not Definitely not Unsure Prefer not to say	‘Definitely yes’ was reclassified as ‘1’. ‘Prefer not to say’ was reclassified as ‘No data’. All other responses were reclassified as ‘0’.
Number of COVID-19 vaccine doses received	How many doses of COVID-19 vaccine have you received? State the total number of doses across all vaccine types (AstraZeneca, Pfizer, Moderna, etc.)	I am not vaccinated 1 dose 2 doses 3 doses 4 doses 5 doses >5 doses Unsure/don’t know	I am not vaccinated’, ‘1 dose’ and ‘2 doses’, ‘3 doses’ were collapsed into one category: ‘≤3 doses’. ‘4 doses’, ‘5 doses’ and ‘>5 doses’ were collapsed into one category: ‘>3 doses’. ‘Unsure/don’t know’ was reclassified as ‘No data’.
Perceived susceptibility of COVID-19	How likely do you believe it is that you will be infected with COVID-19 at some point in the future?	Very likely Somewhat likely Unlikely Prefer not to say Not applicable—Have already been infected with COVID-19 Don’t know	‘’Very likely’ was reclassified as ‘1’. ‘Prefer not to say’, ‘Not applicable’, ‘Don’t know’ were reclassified as ‘No data’. All other responses were reclassified as ‘0’.
Perceived severity of COVID-19	If you were infected with COVID-19 in the future, how severe do you think it would be for your health?	Life threatening Very severe (e.g., requiring hospitalization) Moderate (e.g., requiring self-care and rest in bed) Mild (e.g., capable of continuing with daily tasks normally) No symptoms Prefer not to say Don’t know	Life threatening’, ‘very severe’ were reclassified as ‘1’. ‘Prefer not to say’, ‘Don’t know’ were reclassified as ‘No data’. All other responses were reclassified as ‘0’.

**Table 2:** Basic demographic data of Optimise participants. Bold subheadings within the table indicate different demographic categories.

Demographic	Number	% Total (/779)
**Age**		
18–24	46	6%
25–34	198	25%
35–44	137	18%
45–54	116	15%
55–64	131	17%
65+	147	19%
No data	4	0%
**Healthcare workers**		
Yes	163	21%
No	616	79%
Chronic health condition increasing risk of COVID-19 *		
Yes	197	25%
No	582	75%
**Country of birth**		
Australia	503	65%
Other	276	35%

* A binary variable was inferred denoting the presence of one or more chronic health conditions that put the participant at increased risk of COVID-19. Participants were included in this category if they reported Alzheimer’s/dementia, asthma, autoimmune disease, bowel disease, cancer, chronic kidney conditions, chronic lung conditions, diabetes, heart disease, hypertension/high blood pressure, immune disorders, liver conditions, musculoskeletal conditions or stroke.

**Table 3:** GEE results for associations between intention to receive a first COVID-19 vaccine, perceived severity and perceived susceptibility over time. The reference time (intercept), T1, is 21-Oct-20 to 16-Nov-20.

Variable	Odds Ratio (OR)	95% Confidence Interval	*p*-Value
Main effects			
(Intercept)	2.05	(1.44, 2.92)	<0.01
Perceived severity of COVID-19	1.34	(0.65, 2.79)	0.43
Perceived susceptibility to COVID-19	0.14	(0.01, 1.50)	0.10
Time T2 (17-Nov-20 to 13-Dec-20)	0.89	(0.67, 1.18)	0.43
Time T3 (14-Dec-20 to 09-Jan-21)	0.97	(0.66, 1.41)	0.86
Time T4 (10-Jan-21 to 05-Feb-21)	0.97	(0.69, 1.36)	0.85
Time T5 (06-Feb-21 to 04-Mar-21)	1.35	(0.93, 1.97)	0.11
Time T6 (05-Mar-21 to 31-Mar-21)	1.46	(0.97, 2.21)	0.07
Time T7 (01-Apr-21 to 27-Apr-21)	1.06	(0.71, 1.58)	0.78
Time T8 (28-Apr-21 to 24-May-21)	1.27	(0.84, 1.91)	0.26
Time T9 (25-May-21 to 20-Jun-21)	2.75	(1.81, 4.17)	<0.01
Time T10 (21-Jun-21 to 17-Jul-21)	2.96	(1.95, 4.51)	<0.01
Time T11 (18-Jul-21 to 13-Aug-21)	4.56	(2.81, 7.4)	<0.01
Time T12 (14-Aug-21 to 09-Sep-21)	6.15	(3.77, 10.05)	<0.01
Interactions: Time × Perceived severity			
Time T2 × Perceived severity	0.94	(0.33, 2.66)	0.91
Time T3 × Perceived severity	1.09	(0.59, 2.03)	0.79
Time T4 × Perceived severity	0.78	(0.37, 1.66)	0.53
Time T5 × Perceived severity	0.66	(0.28, 1.57)	0.35
Time T6 × Perceived severity	0.58	(0.24, 1.39)	0.22
Time T7 × Perceived severity	0.68	(0.25, 1.87)	0.46
Time T8 × Perceived severity	0.59	(0.24, 1.45)	0.25
Time T9 × Perceived severity	0.69	(0.29, 1.60)	0.38
Time T10 × Perceived severity	1.45	(0.55, 3.81)	0.46
Time T11 × Perceived severity	0.70	(0.23, 2.11)	0.52
Time T12 × Perceived severity	1.67	(0.17, 16.4)	0.66
Interactions: Time × Perceived susceptibility			
Time T2 × Perceived susceptibility	3.42	(0.49, 23.85)	0.22
Time T3 × Perceived susceptibility	19.86	(0.34, 1157.45)	0.15
Time T4 × Perceived susceptibility	9.05	(0.66, 124.52)	0.10
Time T5 × Perceived susceptibility	3.20	(0.26, 39.99)	0.37
Time T6 × Perceived susceptibility	7.96	(0.75, 84.97)	0.09
Time T7 × Perceived susceptibility	13.93	(1.17, 166.26)	0.04
Time T8 × Perceived susceptibility	8.43	(0.75, 95.01)	0.08
Time T9 × Perceived susceptibility	7.07	(0.55, 90.38)	0.13
Time T10 × Perceived susceptibility	8.94	(0.74, 107.78)	0.08
Time T11 × Perceived susceptibility	7.67	(0.64, 91.81)	0.11
Time T12 × Perceived susceptibility	10.38	(0.86, 125.54)	0.07

**Table 4:** GEE results for associations between intention to receive further COVID-19 vaccine doses, perceived severity and perceived susceptibility over time. The reference time (intercept), T22, is 11 May 2022 to 6 June 2022. Numbering of time periods was set at 22 for this sample to indicate the number of time periods elapsed between data in this analysis and those in Table 3.

Variable	Odds Ratio (OR)	95% Confidence Interval	*p*-Value
Main effects			
(Intercept)	1.58	(1.24, 2.03)	<0.01
Perceived severity of COVID-19	2.53	(1.26, 5.07)	0.01
Perceived susceptibility to COVID-19	1.12	(0.84, 1.51)	0.43
Time T23 (07-Jun-22 to 03-Jul-22)	0.99	(0.77, 1.29)	0.96
Time T24 (04-Jul-22 to 30-Jul-22)	0.92	(0.71, 1.19)	0.53
Time T25 (31-Jul-22 to 26-Aug-22)	1.05	(0.80, 1.39)	0.72
Interactions: Time × Perceived severity			
Time T23 × Perceived severity	0.77	(0.51, 1.17)	0.22
Time T24 × Perceived severity	0.48	(0.26, 0.91)	0.02
Time T25 × Perceived severity	0.54	(0.28, 1.07)	0.08
Interactions: Time × Perceived susceptibility			
Time T23 × Perceived susceptibility	1.06	(0.72, 1.55)	0.76
Time T24 × Perceived susceptibility	1.31	(0.90, 1.90)	0.16
Time T25 × Perceived susceptibility	0.78	(0.53, 1.16)	0.22

**Table 5:** GEE results for associations between vaccine self-reported uptake (having received > 3 doses), perceived severity and perceived susceptibility over time. The reference time (intercept), T22, is 11 May 2022 to 6 June 2022. Numbering of time periods was set at 22 for this sample to indicate the number of time periods elapsed between data in this analysis and those in Table 3.

Variable	Odds Ratio (OR)	95% Confidence Interval	*p*-Value
Main effects			
(Intercept)	0.16	(0.11, 0.21)	<0.01
Perceived severity of COVID-19	2.74	(1.58, 4.76)	<0.01
Perceived susceptibility to COVID-19	0.71	(0.48, 1.04)	0.08
Time T23 (07-Jun-22 to 03-Jul-22)	1.47	(1.12, 1.93)	<0.01
Time T24 (04-Jul-22 to 30-Jul-22)	2.59	(1.90, 3.52)	<0.01
Time T25 (31-Jul-22 to 26-Aug-22)	4.72	(3.36, 6.64)	<0.01
Interactions: Time × Perceived severity			
Time T23 × Perceived severity	0.99	(0.56, 1.74)	0.97
Time T24 × Perceived severity	0.57	(0.33, 0.99)	0.04
Time T25 × Perceived severity	0.63	(0.32, 1.22)	0.17
Interactions: Time × Perceived susceptibility			
Time T23 × Perceived susceptibility	1.04	(0.68, 1.60)	0.86
Time T24 × Perceived susceptibility	1.33	(0.85, 2.07)	0.21
Time T25 × Perceived susceptibility	1.40	(0.87, 2.24)	0.16

**Table 6:** Coefficients and corresponding *p*-values for GEE analysis of perceived severity of COVID-19. The reference category (intercept) is aged 18–24; does not have a chronic health condition and employed full-time. Time is treated a continuous variable with four-week increments, with 21 October 2020 to 16 November 2020 = 1.

Coefficient	Odds Ratio (OR)	95% Confidence Interval	*p*-Value
Intercept	0.09	(0.04, 0.18)	<0.01
Time	0.93	(0.91, 0.95)	<0.01
Age (25–34)	1.08	(0.47, 2.52)	0.85
Age (35–44)	2.25	(1.03, 4.92)	0.04
Age (45–54)	4.14	(1.93, 8.89)	<0.01
Age (55–64)	4.53	(2.15, 9.53)	<0.01
Age (65+)	2.86	(1.33, 6.14)	0.01
Chronic health condition (yes)	2.32	(1.57, 3.43)	<0.01
Employment (not employed or other)	2.12	(1.30, 3.46)	<0.01
Employment (part-time or casual)	1.03	(0.64, 1.65)	0.89

**Table 7:** Coefficients and corresponding *p*-values for GEE analysis of perceived susceptibility of COVID-19. The reference categories (intercept) are aged 18–24; does not have a chronic health condition; employed full-time, is not a healthcare worker, has an income under 50 k and lives in metro Victoria. Time is treated a continuous variable with four-week increments, with 21 October 2020 to 16 November 2020 = 1.

Coefficient	Odds Ratio (OR)	95% Confidence Interval	*p*-Value
Intercept	0.02	(0.01, 0.04)	<0.01
Time	1.16	(1.14, 1.18)	<0.01
Age (25–34)	1.15	(0.72, 1.84)	0.55
Age (35–44)	0.98	(0.59, 1.63)	0.95
Age (45–54)	0.96	(0.58, 1.60)	0.89
Age (55–64)	0.88	(0.54, 1.43)	0.60
Age (65+)	0.54	(0.32, 0.92)	0.02
Chronic health condition (yes)	1.32	(1.03, 1.71)	0.03
Employment (not employed or other)	0.84	(0.59, 1.19)	0.32
Employment (part-time or casual)	1.23	(0.92, 1.66)	0.15
Healthcare worker (yes)	1.31	(0.98, 1.76)	0.07
Income (AUD50–99 k)	1.21	(0.85, 1.72)	0.29
Income (AUD100–149 k)	1.60	(1.15, 2.23)	<0.01
Income (AUD150 k+)	2.20	(1.52, 3.20)	<0.01
Member of religious group (yes)	0.67	(0.45, 0.99)	0.05
Region of residence (regional Victoria)	1.48	(1.08, 2.02)	0.02

**Figure A1:**
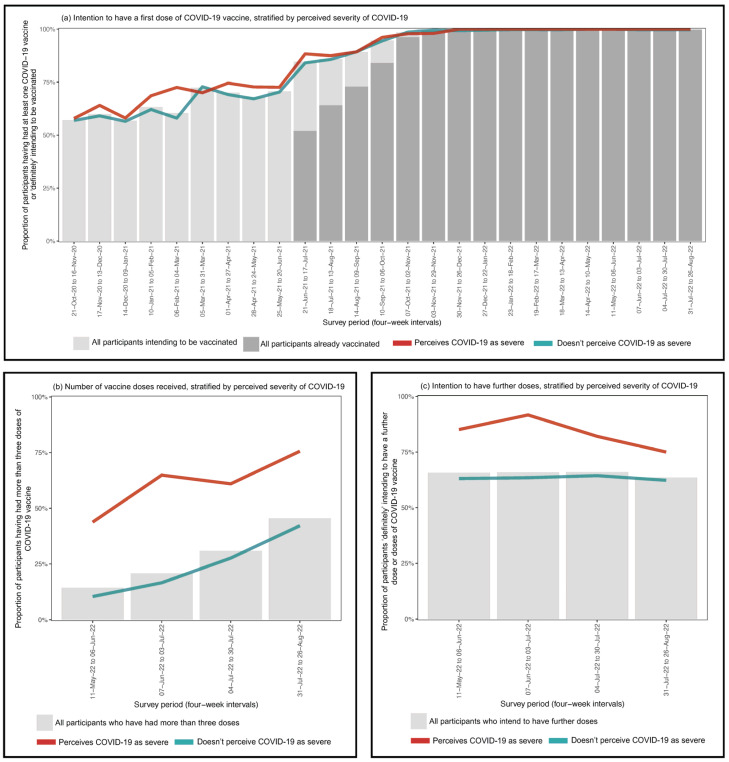
COVID-19 vaccine uptake and intention by perceptions of COVID-19 severity. Red lines represents the proportion of those that perceived COVID-19 as severe that (**a**) intended to receive a first dose of COVID_19 vaccine, (**b**) received more than three doses of the COVID-19 vaccine, and (**c**) intended to have further doses of the COVID-19 vaccine. Green lines represent those that did not perceive COVID-19 as severe that (**a**) intended to receive a first dose of COVID-19 vaccine (**b**) received more than three doses of the COVID-19 vaccine, and (**c**) intended to have further doses of the COVID-19 vaccine.

**Figure A2:**
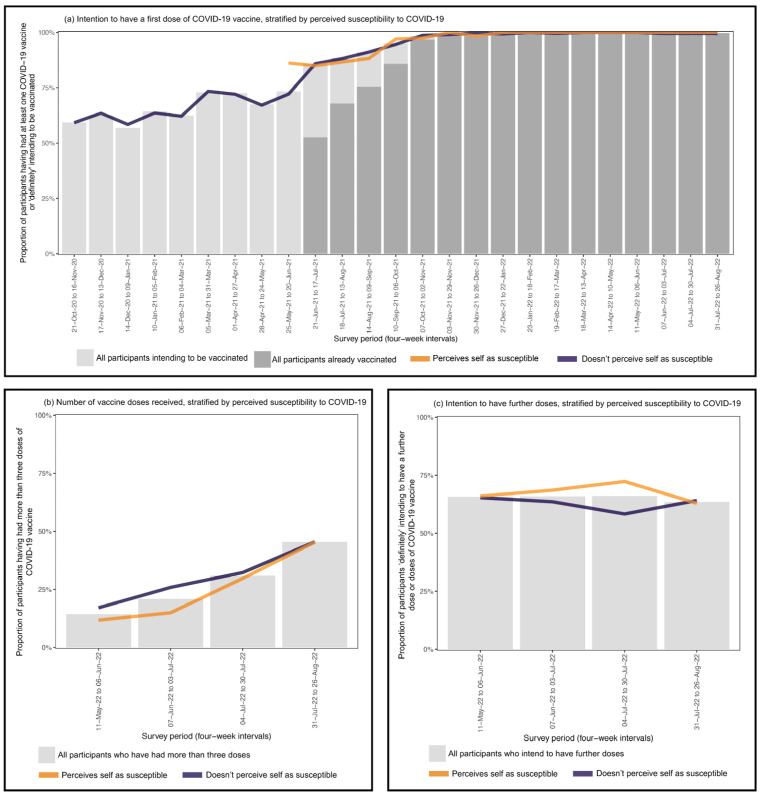
COVID-19 vaccine uptake and intention by perceptions of COVID-19 susceptibility. Orange lines represent those that perceived themselves as susceptible to COVID-19 that (**a**) intended to receive a first dose of COVID-19 vaccine, (**b**) received more than three doses of the COVID-19 vaccine, and (**c**) intended to have further doses of the COVID-19 vaccine. Purple lines represent those that did not perceive themselves as susceptible to COVID-19 that (**a**) intended to receive a first dose of COVID-19 vaccine, (**b**) received more than three doses of the COVID-19 vaccine, and (**c**) intended to have further doses of the COVID-19 vaccine.

## Data Availability

The data presented in this study may be requested from the corresponding author. The data are not publicly available due to ethical considerations and data privacy restrictions.

## Abbreviations

The following abbreviations are used in this manuscript:

GEE	Generalized Estimating Equations
AR	Autoregressive
QIC	Quasilikelihood Independence Model Criterion

## Appendix A

### Demographics

Table A1 presents additional demographic characteristics of the Optimise sample to complement data provided in Table 1 in the main text. Variables include participants’ highest level of education, presence of children living at home, primary language spoken at home, employment status, gender identity, whether they identify as an active member of a religious institution and place of residence (metropolitan or regional).

**Table A1:** Additional demographics of Optimise participants. Bold subheadings within the table indicate different demographic factors.

Demographic	Number	% Total (/779)
**Highest level of education obtained**		
Primary school	39	5%
High school	104	13%
Tertiary education—postgraduate	213	27%
Tertiary education—TAFE/trade certificate	126	16%
Tertiary—undergraduate	290	37%
No data	7	1%
**Participant has children living in their household**		
Yes	217	28%
No	562	72%
**Language spoken at home**		
English	638	82%
Other	141	18%
**Employment status pre-COVID-19 (March 2020)**		
Full-time or self-employed	281	36%
Part-time or casual	271	35%
Not employed or other	227	29%
**Gender identity**		
Female	559	72%
Male	219	28%
No data	1	0%
**Active member of a religious group/church**		
Yes	92	12%
No	675	87%
No data	12	2%
**Area of residence in Victoria**		
Metropolitan Melbourne	628	81%
Regional Victoria	144	18%
No data	7	1%

## Appendix B

### COVID-19 Vaccine Uptake and Intention by Perceived Severity of Infection and Susceptibility of Contracting the Virus

Figure A1 presents trends in (a) intention to have a first COVID-19 vaccine dose, (b) COVID-19 vaccine uptake, and (c) intention to receive further doses, stratified by participants’ perceptions of the severity of COVID-19. Those who perceived the virus as severe (red line) were consistently more likely to have received more than three doses of the vaccine (Figure A1b) and to express an intention to receive further doses (Figure A1c), compared to those who did not perceive COVID-19 as severe (green line). This pattern was less evident for people’s intention to receive a first dose of the vaccine (Figure A1a).

In Figure A2 individuals who felt they were at risk of contracting COVID-19 (orange line) showed marginally lower rates of having received more than three vaccine doses (Figure A2b) and were marginally more likely to intend to receive further doses (Figure A2c), compared to those who did not consider themselves susceptible (purple line). As in the case of perceived severity, this was no visually distinguishable difference in intention to have a first dose the vaccine between those that perceived themselves susceptible to COVID-19 infection and those that did not (Figure A2a). As shown in Table 3, Table 4 and Table 5 in the main text, perceived susceptibility was not significantly associated with any of the three outcomes.
